# Effect of the combination of automated peripheral mechanical stimulation and physical exercise on aerobic functional capacity and cardiac autonomic control in patients with Parkinson’s disease: a randomized clinical trial protocol

**DOI:** 10.1186/s13063-021-05177-w

**Published:** 2021-04-06

**Authors:** Nicolle Zelada-Astudillo, Vinicius Christianini Moreno, Andrea Herrera-Santelices, Fabio Augusto Barbieri, Antonio Roberto Zamunér

**Affiliations:** 1 Laboratory of Clinical Research in Kinesiology, Department of Kinesiology, Universidad Católica del Maule, Talca, Chile; 2 Servicio de Medicina Física y Rehabilitación, Hospital San Juan de Dios, Curicó, Chile; 3 São Paulo State University (UNESP), Graduate Program in Movement Sciences, Department of Physical Education, Human Movement Research Laboratory (MOVI-LAB), Bauru, Brazil

**Keywords:** Aged, Parkinson’s disease, Rehabilitations, Exercise, Randomized controlled trial

## Abstract

**Background:**

Automated peripheral mechanical stimulation (AMPS) has been proposed as a new complementary therapy with potential for improving motor and cardiovascular abnormalities in Parkinson’s disease (PD). However, AMPS long-term effects and its combination with physical exercise are unknown. Thus, this study aims to compare the effects of a program of 12 weeks of physical exercise with a 12-week intervention program combining physical exercise and AMPS on the aerobic capacity, cardiac autonomic control, and gait parameters in patients with PD.

**Methods:**

A randomized, controlled clinical trial will be conducted. Older volunteers with PD will be randomly assigned to one of the two groups studied: (1) exercise or (2) AMPS + exercise. Both groups will undergo an exercise program of 24 sessions, for 12 weeks, performed twice a week. Before exercise sessions, the group AMPS + exercise will receive a session of active AMPS, while the group exercise will receive an AMPS sham intervention. Shapiro-Wilk’s and Levene’s tests will be used to check for data normality and homogeneity, respectively. In case parametric assumptions are fulfilled, per-protocol and intention-to-treat analyses will be performed using a mixed model analysis of variance to check for group*time interaction. Significance level will be set at 5%.

**Discussion:**

Several non-pharmacological treatment modalities have been proposed for PD, focusing primarily on the reduction of motor and musculoskeletal disorders. Regular exercise and motor training have been shown to be effective in improving quality of life. However, treatment options in general remain limited given the high prevalence and adverse impact of these disorders. So, developing new strategies that can potentiate the improvement of motor disabilities and also improve non-motor symptoms in PD is relevant. It is expected that the participants from both groups will improve their quality of life, gait parameters, and their cardiac autonomic control, with greater improvements being observed in the group combining active AMPS and physical exercise.

**Trial registration:**

ClinicalTrials.gov NCT04251728. Registered on February 05, 2020.

**Supplementary Information:**

The online version contains supplementary material available at 10.1186/s13063-021-05177-w.

## Administrative information

Note: the numbers in curly brackets in this protocol refer to SPIRIT checklist item numbers (Additional file 1). The order of the items has been modified to group similar items (see http://www.equator-network.org/reporting-guidelines/spirit-2013-statement-defining-standard-protocol-items-for-clinical-trials/).

**Table:** 

Title {1}	Effect of the combination of automated peripheral mechanical stimulation and physical exercise on aerobic functional capacity and cardiac autonomic control in patients with Parkinson’s disease: a randomized clinical trial protocol.
Trial registration {2a and 2b}.	ClinicalTrials.gov, NCT04251728, registered February 05, 2020
Protocol version {3}	November 01, 2021. Version 4.
Funding {4}	National Fund for Scientific and Technological Development of Chile (FONDECYT).
Author details {5a}	**Nicolle Zelada-Astudillo (NZA)** Universidad Católica del Maule, Talca, Chile. **Andrea Herrera-Santelices (AHR):** Universidad Católica del Maule, Talca, Chile. Servicio de Medicina Física y Rehabilitación, Hospital San Juan de Dios, Curicó, Chile. **Fabio Augusto Barbieri** São Paulo State University (UNESP), Graduate Program in Movement Sciences, Department of Physical Education, Human Movement Research Laboratory (MOVI-LAB), Bauru, Brazil. **Vinicius Christianini Moreno** São Paulo State University (UNESP), Graduate Program in Movement Sciences, Department of Physical Education, Human Movement Research Laboratory (MOVI-LAB), Bauru, Brazil. **Antonio Roberto Zamunér (ARZ):** Laboratory of Clinical Research in Kinesiology, Department of Kinesiology, Universidad Católica del Maule, Talca, Chile.
Name and contact information for the trial sponsor {5b}	CONICYT-FONDECYT (Fondo Nacional de Desarrollo Científico y Tecnológico) Phone: +56 2 2365 4400
Role of sponsor {5c}	Grant #11180310: Provide funding to develop the project.

## Introduction

### Background and rationale {6a}

Parkinson’s disease (PD) is a degenerative neurological disorder characterized by a loss of dopaminergic neurons at the substantia nigra level, leading to muscle tremor, rigidity, progressive bradykinesia, and postural instability, with great impact on the quality of life [1]. Based on international data, it can be estimated that there are more than seven million patients with PD worldwide [2]. Moreover, about 1 to 2% of the population over 65 years of age suffers from PD, rising to 3 to 5% in those over 85 years of age [3].

Gait disorders are one of the main therapeutic challenges in PD [4], involving a risk of fall, disability, and physical decline [5]. The progressive deterioration of the physical condition leads to a reduction in aerobic functional capacity and autonomy, thus affecting the ability to perform everyday tasks effectively [6, 7]. Indeed, it is estimated that between 40 and 70% of people with PD have falls, in which muscle weakness at lower limbs appears to play a key role, significantly reducing their postural stability [8]. These data allow us to consider elderly people with PD in a high degree of frailty, thus with high probability of suffering health problems due to the massive deterioration of physiological systems [9, 10].

In terms of intrinsic exercise capacity, studies have shown that the maximum oxygen uptake (VO_2_) of PD patients was no different from that of controls, but they reached their maximum VO_2_ earlier, suggesting a lower mechanical efficiency of movement during exercise, perhaps due to their muscle stiffness [11]. Moreover, some authors have studied the relationship between aerobic functional capacity and gait [12, 13]. Results showed a positive correlation between VO_2peak_ and gait speed, meaning that the lower the cardiorespiratory capacity the lower the gait speed.

Besides motor abnormalities, patients with PD present non-motor symptoms such as dysautonomia [14] and sleep disturbances [15]. Thus, physical exercise has been highly recommended as non-pharmacological intervention since it has shown to attenuate motor dysfunction, improve cognitive function, effectiveness of medications, and sleep patterns, as well as prevent depression and cardiovascular complications [16]. In this sense, Amara et al. mention that regular practice of physical exercise has positive effects on the effectiveness of sleep in the long term for patients with PD [17]. Furthermore, according to Fan et al. [18], physical exercise, especially those performed at moderate to vigorous intensities, has a positive impact on PD through multiple mechanisms, including reduction of α-synuclein protein accumulation, decrease in inflammation and oxidative stress, improvement in brain-derived neurotrophic factor (BDNF) activity, nerve regeneration, and mitochondrial function. Furthermore, some authors have shown that endurance exercise is safe and well-tolerated for people with PD from a cardiovascular point of view, which supports their recommendation for this population [19].

Although physical exercise has shown to be effective, some authors have proposed other non-pharmacological strategies as complementary therapy. In this sense, Barbic et al. [20] proposed an innovative approach aiming at improving walking parameters in PD. The authors evaluated the effect of a single session of peripheral mechanical stimulation therapy. After the stimulus, the authors observed significant changes in motor parameters such as improvement in gait length and gait speed. Concomitant to the motor improvement, surprisingly, there was an improvement in some cardiovascular parameters, such as decreased resting blood pressure and improved cardiovascular autonomic control in response to orthostatic stimulation. In addition, Zamunér et al. [21] studied the effects of five sessions of automated mechanical peripheral stimulation (AMPS) on cardiovascular parameters in patients with PD. The authors reported that after the AMPS therapy, patients with PD presented a decrease in cardiovascular sympathetic control and an improvement in arterial baroreflex sensitivity.

AMPS therapy consists of applying a mechanical pressure with intensity to elicit pain (at pain pressure threshold) at four specific points on the sole of both feet of patients with PD. Moreover, the total therapy duration is 96 s, and some studies have reported that the effects may last up to 72 h [22, 23]. Therefore, due to its promising beneficial results and that it is not a time-consuming therapy, AMPS represents an interesting intervention strategy. So, considering that AMPS therapy promotes beneficial motor and cardiovascular effects for PD patients in the short term, as well as physical exercise, it is important to study whether adding AMPS to an exercise rehabilitation program could potentiate the effects of exercise, since patients are expected to attend the sessions with the potential for improved performance and execution of the exercises during the sessions. Furthermore, no studies have addressed the long-term effects of AMPS (i.e., AMPS intervention program lasting more than 1 month) in patients with PD.

### Objectives {7}

The objectives of this study will be to evaluate the effect of 24 sessions of AMPS combined to a physical exercise program performed twice a week for 12 weeks on aerobic functional capacity, gait parameters, and cardiac autonomic control in patients with PD. As a hypothesis, the exercise program combined with AMPS therapy is expected to be more effective in improving cardiac autonomic control and aerobic functional capacity in PD patients than exercise alone.

### Trial design {8}

The present study is a two-arm parallel double-blind randomized controlled trial. The sample will be selected on a non-probability basis. Participants will be randomly assigned in a 1:1 ratio to one of the two groups: (1) exercise group (EX-G) or (2) group combining AMPS and exercise (AMPS-G).

## Methods: participants, interventions, and outcomes

### Study setting {9}

The study will be carried out at the Laboratory of Clinical Research in Kinesiology from the Universidad Católica del Maule, Talca, Maule, Chile and Human Movement Research Laboratory (MOVI-LAB), Bauru, Brazil.

### Eligibility criteria {10}

#### Inclusion criteria

Volunteers must present a clinical diagnosis of PD, made by a board-certified neurologist, according to the UK Brain Bank criteria [24] aged 50 years or over and be classified as 1 to 3 at the Hoehn and Yahr scale. Drug treatment of volunteers should remain unchanged for at least 30 days prior their inclusion in the study.

#### Exclusion criteria

Volunteers who show signs of cognitive impairment, based on the results of the Mini-Mental State Examination adjusted for the educational level of the Chilean and Brazilian population [25], who present cardiorespiratory, neuromuscular, and musculoskeletal diseases will be excluded. Volunteers who have peripheral sensory neuropathy, diabetes, or any other condition or disease known to promote autonomic dysfunction will be excluded based on symptoms, physical condition, and routine laboratory tests.

### Who will take informed consent? {26a}

NZA researcher will be in charge to contact potential participants via telephone and invite them to participate in the study. In case we have a positive reply, an appointment will be scheduled and NZA will explain the objectives of the study, the inclusion and exclusion criteria, participant’s roles, risks, benefits, and ethical implications. If the participants agree in took part in the study, they will be requested to sign two copies of an informed consent, one for the participants and the other for the researcher.

### Additional consent provisions for collection and use of participant data and biological specimens {26b}

Not applicable, this trial does not have biological specimens.

### Interventions

#### Explanation for the choice of comparators {6b}

In order to comply with the proposed objective and verify whether AMPS therapy improves exercise performance by enhancing its benefits and taking into account a possible placebo effect, participants will be randomly assigned to one of the two studied groups: (1) exercise group (EX-G): participants will undergo 12-week exercise program, held twice a week along with 2 weekly sessions of inactive SHAM during the same period; (2) group combining AMPS and Exercise (AMPS-G): participants in this group will undergo the same 12-week exercise program of the EX-G, along with 2 weekly sessions of active AMPS during the same period.

#### Intervention description {11a}

##### Automated mechanical peripheral stimulation

AMPS therapy will be applied through a therapeutic device (Gondola, Ecker Technologies, Lugano, Switzerland), which will be fitted to each patient’s feet. The device consists of a foot stand for each foot with two electric motors that activate two 2-mm-diameter metal bars. The mechanical pressure stimulus occurs when the motors are activated. Before application, the device is adjusted so that the stimulus is applied to two specific areas for each foot, at the base of the first metatarsal joint and at the head of the hallux of the right and left feet. The intensity of the pressure is adjusted to reach the participant’s pain threshold (which normally occurs around 0. 3–0. 9 N mm^2^), defined as the moment when the participant reports that they began to feel pain (the moment of transition between the strong pressure and the painful pressure and the first time the visual analog scale (VAS) is greater than 0) or at the moment when the monosynaptic reflex appears in the anterior tibial muscle. After these preparatory procedures, treatment begins. The therapy will be carried out in 4 cycles. A cycle is completed when the four target areas are stimulated, which requires 24 s (6 s per area) so the complete therapy lasts 96 s [26].

The SHAM intervention will replicate the same procedure described for active AMPS. However, a rubber adapter will be positioned at the tip of the metal bars to increase the contact surface area and the pressure will be set to the sensorial threshold (i.e. when the patient report that they feel a touch at the point). AMPS sessions will be performed 24 h before all the exercise sessions [27].

##### Physical exercise program

Physical exercise sessions will be composed of:

1. Warm-up (5 min): patients perform stretching of the main muscle groups of the upper limbs, lower limbs, and trunk.
2. Aerobic exercise (30 min): Patients will perform continuous aerobic exercise consisting of walking on flat ground and ramps, with intensity between 40 and 60% of the heart rate reserve.
3. Strengthening exercises (20 min): volunteers will perform resistance exercises (2 series × 15 repetitions) for the upper and lower limbs, and the trunk working the following muscle groups: shoulder flexors, extensors, and abductors; elbow flexors and extenders; trunk extensors and flexors; knee flexors and extenders; and dorsiflexes and plantar flexors.
4. Cool down (5 min): Stretching of the main muscle groups worked during the sessions and relaxation.

After 6 weeks and in the end of the intervention program, the participants will be asked to inform which kind of intervention they thought they had been on (i.e., placebo or active) and to report their confidence level rating on a scale of 0–10, with 0 meaning “not at all confident” and 10 “extremely confident” that they received the active therapy [28].

#### Criteria for discontinuing or modifying allocated interventions {11b}

Participants who attend less than 70% of the training sessions, participants who have been absent on evaluation days and participants who start performing other regular physical exercises after baseline assessments will be excluded from the study.

#### Strategies to improve adherence to interventions {11c}

In order to improve adherence to interventions, researchers will continuously request feedback from the participants throughout the sessions to check for possible discomfort and adjust the exercises execution if it is needed. Moreover, every session will be supervised by at least two researchers with experience in exercise rehabilitation, to ensure the patients are executing the exercises properly.

#### Relevant concomitant care permitted or prohibited during the trial {11d}

Participants will be allowed to keep their pharmacological treatment during the entire study. However, participants will be excluded in case of any dose modification during the trial. In addition, participants may continue to attend physiotherapy interventions and any other non-pharmacological interventions if it is a regular practice started at least 6 months before the study. In this case, participants will be requested to maintain this activity during the entire study; otherwise, they will be excluded.

However, they will be excluded in case of suspension of any intervention or start of a new regular intervention. For the outcome assessments performed at baseline and after the intervention program, participants will be instructed not to drink alcohol and/or stimulants (coffee, teas, and energy drinks), avoiding heavy meals, having a light meal at least 2 h before the test and not doing strenuous exercise 48 h before the evaluations.

#### Provisions for post-trial care {30}

At the end of the study, if AMPS is effective in promoting higher improvement than exercise alone, the same treatment will be offered at no cost to EX-G participants.

#### Outcomes {12}

The results will be evaluated at 2 time points: (1) baseline (before starting the intervention program) and (2) after 12 weeks of intervention (after 48 h to 1 week from the last intervention). The primary outcomes will be (1) quality of life (PDQ-39), (2) heart rate variability (HRV) indices, and (3) VO2peak (cardiopulmonary exercise testing). Secondary outcomes include (1) 24-h blood pressure (3 channel Cardio Map digital equipment), (2) sleep quality (actigraph monitor), (3) VO2 at the anaerobic threshold (cardiopulmonary exercise testing), (4) dynamic balance (Tinetti Scale, TUG), and (5) gait velocity (10-m Walk Test).

#### Participant timeline {13}

**Fig. 1:**
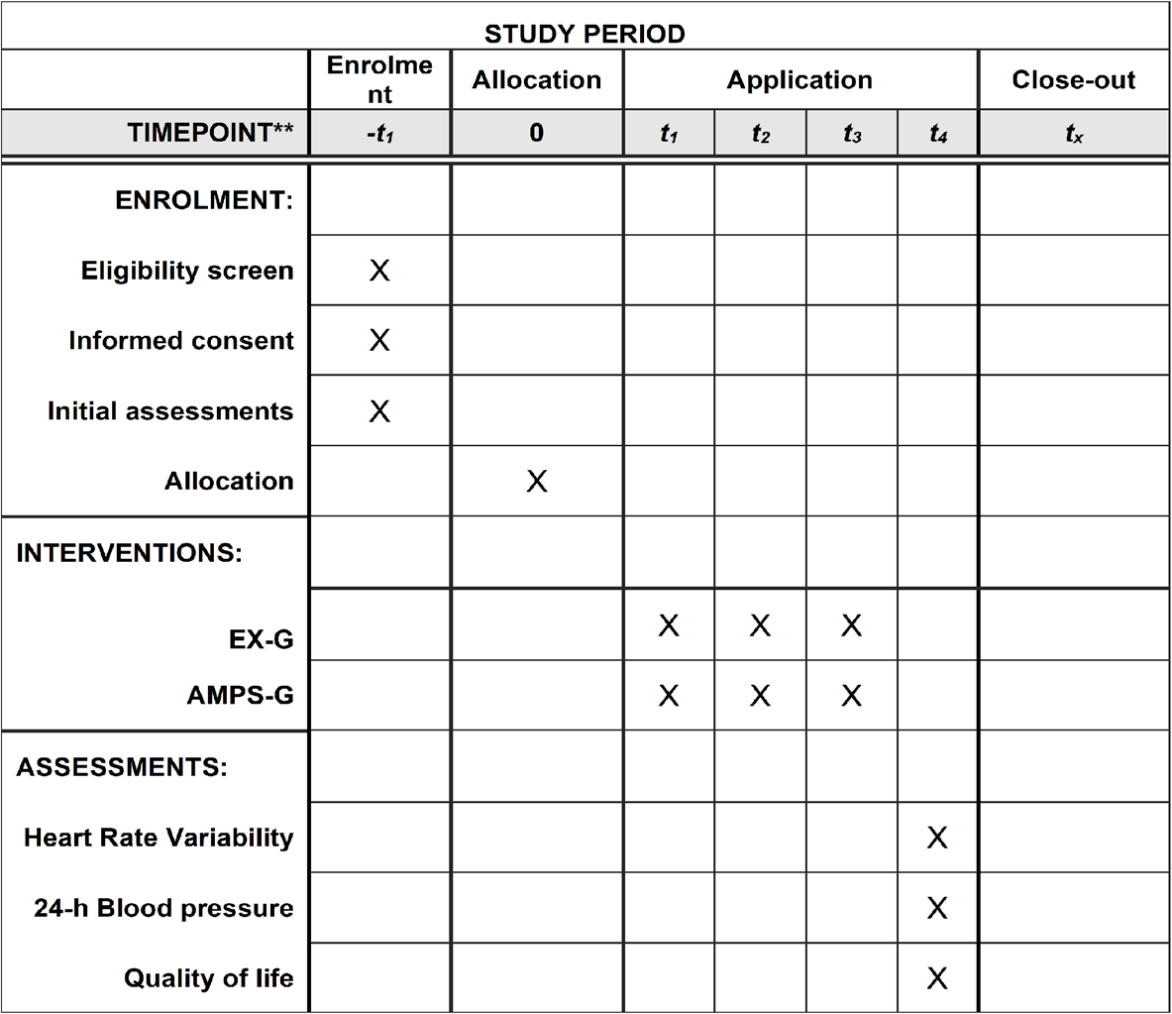
The recommended SPIRIT figure with the participant timeline

Figure 1 show the recommended SPIRIT figure with the participant timeline.

#### Sample size {14}

To determine the number of volunteers, the sample size was calculated using the GPower software version 3.1, for a 95% confidence level and 80% study power. The calculation was based on a pilot study (unpublished) that considered the LF/HF index of HRV, and systolic blood pressure. The largest suggested sample size was 19 volunteers per group to detect a decrease in systolic blood pressure with an effect size of 0.80. Therefore, considering possible dropouts, 25 participants will be enrolled for each group.

#### Recruitment {15}

Participants will be recruited from the community by radio and online advertisements and notices, advertisements posted in strategic places such as neurological clinics. Also, researchers will contact the director of all Public Family Health Center (CESFAM) in the region of Maule to present the project and to check for potential participants. If a participant has no confirmed diagnosis of Parkinson’s disease, he/she will be referred to a board-certified neurologist who will apply the UK Brain Bank criteria [24].

### Assignment of interventions: allocation

#### Sequence generation {16a}

A researcher not involved in this study will be in charge to generate the sequence number using a computerized algorithm (https://www.graphpad.com/quickcalcs/randomize2/). Participants will be randomly assigned, in a 1:1 ratio, to one of the two groups studied (1) exercise (coded as “A”) or (2) AMPS + exercise (coded as “B”).

#### Concealment mechanism {16b}

After the sequence number is generated, opaque sealed envelopes containing the letters “A” or “B” will be prepared according to the predefined sequence. The envelopes will be kept safe by the same researcher in charge of sequence generation.

#### Implementation {16c}

**Fig. 2:**
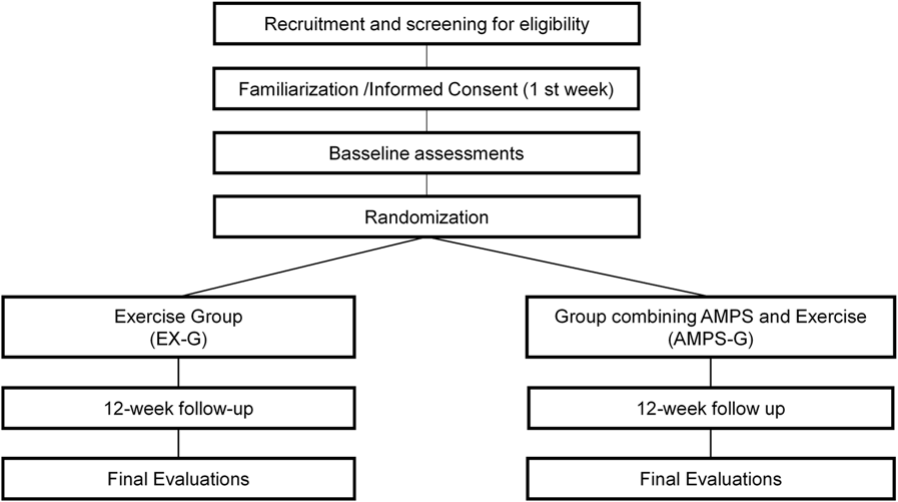
The study design flowchart describing all the steps of the study

Sequence number is generated by a researcher blinded to the study protocol. Opaque envelopes in numerical order will is then prepared and kept in a safe place until baseline assessments are concluded. Participants are recruited in the community, where the study takes place, by all researchers involved. Participants meeting the inclusion criteria proceed to the baseline assessments. Then, prior the beginning of the intervention the envelope labeled with the same number as assigned to the participants when they were included in the study will be unsealed and informed to the leading research. Figure 2 shows the study design flowchart describing all the steps of the study (Fig. 2).

### Assignment of interventions: blinding

#### Who will be blinded {17a}

This will be a double-blinded study. Participants will be assigned to exercise plus AMPS or Exercise plus SHAM group. Thus, participants will be not aware if they are assigned to active AMPS or sham. Also, the researchers responsible for outcome assessments, data entry, and statistical analysis will be blinded to the group assignments. The leading research who will be in charge of the AMPS therapy will not be blinded, since it is not possible.

#### Procedure for unblinding if needed {17b}

In case a participant report to know their intervention group during the trial, it will be immediately communicated to the leading researcher and the participant will be excluded from the study. At the end of the study, in case the treatment is effective, it will be offered to these participants.

### Data collection and management

#### Plans for assessment and collection of outcomes {18a}

Both interventions (AMPS and exercise) will be carried out within the facilities of the Catholic University of Maule, located at Avenida San Miguel 3605, in the city of Talca, in parallel with Human Movement Research Laboratory of the São Paulo State University, Brazil. Evaluations will be conducted in a room with temperature maintained between 22 and 24 °C and relative humidity between 40 and 60% at the same time of day (2:00 p.m. to 6:00 p.m.) to minimize the influences of circadian variations. The methodological procedures for obtaining each outcome are detailed below:

##### Quality of life

Quality of life will be assessed by the Parkinson’s Disease Questionnaire 39 (PDQ-39), which is a questionnaire developed specifically for people with PD and consists of 39 items, and assesses 8 dimensions of daily life, including relationships, social situations, and communication [29]. It is validated for people with PD, has an acceptable internal consistency (α = 0.51 to 0.96), and proved to be reproducible (0.86 to 0.96) [30].

##### Heart rate variability

HRV is defined as the variation that occurs in the time interval between consecutive heartbeats and it has been proposed that its behavior depends on autonomic modulation, as well as its implications on cardiovascular mortality [31]. This time it will be done by continuous recording of RR intervals with 1000 Hz sampling frequency (Polar V800, Finland). R-R intervals will be recorded with a Polar V800 heart rate monitor (Polar, Oi, Finland); a sensor will be placed on the chest at the fifth intercostal space. Prior the beginning of data collection, volunteers will remain at rest for approximately 20 min for baseline heart rate and BP stabilization. Recording will occur under the following conditions: (1) resting in supine position for 15 min and (2) resting in orthostatic posture for 15 min. The volunteers will be instructed to maintain spontaneous breathing throughout the collection period in the supine and orthostatic postures. The participants’ respiratory rate will be recorded throughout the test. Heart rate variability will be analyzed through spectral analysis in an autoregressive model. The spectral components will be obtained at low frequency (LF, 0.04–0.15 Hz) and high frequency (HF, 0.15–0.4 Hz). The LF band is modulated by the sympathetic and parasympathetic autonomic nervous system (with sympathetic predominance), the HF band is associated with cardiac vagal control, and the LF/HF ratio is calculated to assess sympathovagal balance.

##### Peak oxygen uptake

All participants will undergo a cardiopulmonary exercise testing (CPET) studies in a global and non-invasive way the integral response of the organism to exercise, through a rational analysis of the respiratory, cardiovascular, hematopoietic, neuropsychological, and skeletal muscle systems [32] CPET will be performed with an ergometric bike (Ergoselect 5 M, Ergoline, Germany). An incremental ramp-type protocol will be used to determine the workload imposed on aerobic training and possible comorbidities that impede the performance of the proposed physical training protocol [33]. The outcomes VO2, carbon dioxide output (VCO_2_), ventilation (VE), and respiratory exchange ratio (RER) will be obtained on a breath-to-breath basis during the entire CPET using an expired gas measurement system (metalyzer 3b - Cortex Medical, Germany) duly calibrated before each test (this equipment is available for common use in our university). During CPET, electrocardiogram and heart rate (HR) will be continuously recorded in real-time via a 12-lead integrated ECG (Quark T12, Cosmed, Italy). Blood pressure and the ratings of perceived exertion using the Borg CR10 scale will be monitored throughout the test.

##### 24-h blood pressure

The electrocardiogram and blood pressure will be recorded for 24 h concomitantly through the 3 channel Cardio Map digital equipment, before and after the application of the protocol (Cardio Sistemas Comércio e Industria Ltda, Sao Paulo, SP, Brazil).

##### Sleep quality

Sleep quality will be assessed using an actigraph monitor (ActTrust, Condor Instruments Ltda) [34]. Sleep quality will be assessed by quantifying the sleep onset, sleep latency, sleep duration, sleep efficiency, and sleep disturbances. This device has been used in people with sleep disorders, obtaining very good results and demonstrating that it is a low-cost protocol, more durable assessment, and simple in its interpretation, also has good sensitivity and accuracy [35].

##### Timed up and go

It was designed to measure mobility in older people and has been recommended as a useful tool to quantify locomotor performance in people with PD [36]. It is also validated for adults with Parkinson’s with a sensitivity of 76%, a specificity of 66%, a positive predictive value of 39%, and a negative predictive value of 91% [34]. An instrumented TUG (iTUG) will be used to complement the results obtained from the TUG and to characterize the motor impairment resulting from the disease [37].

##### Tinetti scale

Evaluates the mobility of the elderly, the scale has two domains: gait and balance; its main objective is to detect those elderly people at risk of falling, it has a greater predictive value than the muscle test (Tinetti, 1986). It is also validated for adults with PD with a sensitivity of 76%, a specificity of 66%, a positive predictive value of 39%, and a negative predictive value of 91% [38].

##### 10-m walk test

It is a performance measure used to evaluate walking speed in meters per second over a short distance. It can be used to determine functional mobility, gait, and vestibular function [39]. The assessment measures the time in seconds it takes the patient to travel 10 m in a straight line. It will be applied in a corridor with a distance of more than 10 m and a flat surface. This test will be done 3 times conserving the best time that will be registered by 2 evaluators simultaneously.

#### Plans to promote participant retention and complete follow-up {18b}

A weekly phone call will be made to participants in both groups, asking about their health status and reinforcing the pre-session recommendations for physical exercise. Moreover, the participants who wish and agree, will be added to a telephone messenger group app with the objective of solving doubts and share information and experiences about PD.

#### Data management {19}

After inclusion in the study, a unique identification code will be assigned to each participant. So, all data will be collected in an anonym case report form. An Excel file including the individual codes and the corresponding participants will be stored by the leading researcher (AZ) and protected by a password. All data collected/analyzed will be entered into an excel anonymized/coded database by a researcher (NZ or VCM) and double checked by another researcher (AHS or FAB).

All the information gathered from this investigation will always be safeguarded and kept under the care of the Investigator in charge (ARZ), who will allocate a locker in his office under lock and key where all documents of this research will be kept.

All electronic material will be properly stored and backed up by the leading researcher (ARZ), in a computer with a password. Consents and printed documents generated will be stored for 5 years after the end of the study, which will be discarded thereafter.

#### Confidentiality {27}

The confidentiality of the identity will be safeguarded by the following measures:

1. Only the responsible researcher and his or her team will have access to the data collected during the evaluations.
2. The interviews and data will be given a code for each participant, which will only be known by the researcher responsible for this study and will be carried out in an environment that encourages communication and anonymity chosen by the participant.

The data collected will be used only in academic research and research dissemination and will be available to researchers upon a reasonable request to the leading researcher.

#### Plans for collection, laboratory evaluation, and storage of biological specimens for genetic or molecular analysis in this trial/future use {33}

Not applicable, this trial does not have biological specimens.

### Statistical methods

#### Statistical methods for primary and secondary outcomes {20a}

The Shapiro-Wilk and Levene’s tests will be applied to check for data normality and the homogeneity of the variance, respectively. To check the interaction between the Groups (EX-G and AMPS-G) and time (baseline and post-intervention) a mixed ANOVA model with Bonferroni adjustment will be applied for the variables that meet the ANOVA assumptions. Otherwise, the data will be analyzed by the Wilcoxon (intragroup) and Mann-Whitney (intergroup) tests with a priori Bonferroni correction. For all tests, the significance level will be set at 5%.

#### Interim analyses {21b}

Not applicable. Interim analyses will not be performed in the present study.

#### Methods for additional analyses (e.g., subgroup analyses) {20b}

Not Applicable. Additional analyses are not planned in the present study.

#### Methods in analysis to handle protocol non-adherence and any statistical methods to handle missing data {20c}

Per-protocol and intention-to-treat analysis will be performed. Per-protocol analysis will include only participants who attended at least 70% of the sessions and underwent baseline and final evaluations. Missing data will be addressed by a multiple imputation method.

#### Plans to give access to the full protocol, participant level-data and statistical code {31c}

Participants may consult the information generated at any time during the execution of the project upon request to the investigator responsible for the study, who undertakes to provide cooperation and to propose ways to access it.

Likewise, the Responsible Researcher agrees with each participant to send the research report generated at the end of the study to the respective emails, as well as copies of the scientific articles that may result from the study.

### Oversight and monitoring

#### Composition of the coordinating centre and trial steering committee {5d}

This study is proposed by the leading researcher ARZ, coordinator of the Laboratory of Research in Clinical Kinesiology at the Catholic University of Maule. The trial will be directed by the researchers FAB and ARZ. This trial was approved by the scientific ethics committee of the Catholic University of Maule and a does not require the supervision of a steering committee, since there are no invasive procedures, and the assessments and interventions are considered of low risk. Even though, all researchers will meet every 2 weeks to discuss the research progress and possible unforeseen events. Also, all intervention sessions will be supervised by at least two professionals experts in the field of rehabilitation/exercise intervention.

#### Composition of the data monitoring committee, its role and reporting structure {21a}

The protocol excludes vulnerable people and people with diseases that may hinder the application of the protocol. So, it can be considered of low risk and of short duration, thus not requiring an additional committee [40].

The leading researchers (ARZ and FAB) will meet every 2 weeks with all researchers involved in this study to monitor the data, discuss the research progress and any adverse event that arises during the procedures. Researchers are instructed to report any issue to the leading investigators immediately.

#### Adverse event reporting and harms {22}

In the end of each intervention session, participants will be asked to report any complaints and symptoms produced by the proposed activity. Outcome assessor and the lead researcher will be in charge to collect and record this information throughout the study. Adverse events, undesirable effects, and incidents will be classified by specialists as treatment-related or non-treatment-related within 24 h of occurrence [41]. Common undesirable effects may include fatigue, dyspnea, and muscle soreness not lasting more than 24–48 h as a result of the exercise and AMPS intervention. In case these symptoms persist, or other related-treatment event occur, the participants will be asked to discontinue from this study and this will be immediately informed to the ethics committee who will decide whether the protocol should be suspended. All complications and dropouts will be reported in the final manuscript.

#### Frequency and plans for auditing trial conduct {23}

The trial conduct will be continuously monitored by the principal investigators (ARZ and FAB) and it will be discussed in the periodic meetings occurring every 2 weeks with all researchers involved. This trial was approved by the scientific ethics committee of the Catholic University of Maule and since the interventions (i.e. assessments and treatment) are considered of low risk, the supervision by an independent data monitoring committee was not required or considered for this study. However, as aforementioned, the researchers are committed to report any adverse events occurring during the study to the Ethics committee.

#### Plans for communicating important protocol amendments to relevant parties (e.g., trial participants, ethical committees) {25}

In case of any change in the current protocol, the principal investigator will be responsible for reporting and submitting the new version to the ethics committee for approval. After approval, the clinical record will be updated and all modifications will be reported.

#### Dissemination plans {31a}

Each participant will receive a full report with the results of their assessments. By the end of the study, the lead researcher will contact all the participants to provide the final results of the trial.

## Discussion

Several non-pharmacological treatment modalities have been proposed in the treatment of PD, focusing primarily on the treatment of motor and musculoskeletal disorders [42]. In this context, regular exercise and motor training have been shown to be effective in improving people’s quality of life, balance, physical functioning, and activities of daily living, and are therefore strongly recommended for patients with PD [43].

An analysis of the potential benefits of exercise in people with PD concluded that exercise could improve physical functioning, health-related quality of life, leg strength, balance, posture, walking and fitness [44]. Therefore, the authors’ statement that exercise improves functional motor performance in PD patients appears robust; however, the question remains as to which exercise protocol is most appropriate for individual patients [16]. In summary, the evidence base for treating a variety of non-motor symptoms in PD has grown substantially in recent years. However, treatment options in general remain limited given the high prevalence and adverse impact of these disorders, so the development and testing of new treatments for non-motor symptoms in PD remains a priority [45].

In this sense, some authors have proposed the use of AMPS as complementary therapy. This therapy has shown to provide promising effects such as better performance in the gait pattern [37] and decrease the treating freezing of gait [46]. Also, a single session of AMPS treatment is able to modify the resting state in regions of the brain and cerebellum [47], changes the shuffling steps pattern (that is typical of PD subjects), increases the shuffling steps pattern of hip, knee and ankle joints during the gait cycle [23]. In addition, AMPS treatment induces an increased activation of brain areas involved in the visual-space and in the sensory-motor integration [48], which could explain the positive effects on bradykinesia and several gait parameters [49].

Another point that must be mentioned is that patients with PD present disautonomia [50] and high prevalence of cardiovascular diseases [51]. It is well established that exercise is effective in improving cardiac autonomic control, usually quantified by heart rate variability indices, which is considered a prognosis marker for cardiovascular mortality [52, 53]. In the same line, some studies have shown that AMPS may improve the cardiovascular autonomic control [20, 21]. However, despite the positive effects of exercise and AMPS for patients with PD, no studies have addressed its long-term effects combined with an exercise program, since it is a relatively new intervention. Therefore, this study may help to elucidate the potential beneficial effects of adding AMPS to a rehabilitation program. If AMPS provides positive results, it may represent a new therapy strategy for patients with PD complementary to the physical exercise.

Our hypothesis is that the exercise program combined with AMPS therapy would be more effective in improving cardiac autonomic control and aerobic functional capacity in PD patients than exercise alone. This can be explained by two aspects. First, the combination of interventions can change brain functioning strongly than only exercise. While AMPS presents potential to change sensory-motor integration [54], exercise promotes consistent benefits on brain functioning and body system, but without consistent effects on sensory-motor integration. Second, the application of AMPS before the exercise can improve the performance of patients with PD during exercise. The positive effects of AMPS on walking and balance [55] can increase the exercise intensity and quality of movements during the exercise session. Increasing exercise intensity is an important component to improve the benefits of exercise in PD [56]. Thus, in case our hypothesis is confirmed, patients with PD submitted to physical exercise program combined to AMPS could have a greater improvement in their functionality, quality of life and cardiac autonomic control.

### Trial status

Version 4. November 01, 2021.

Date recruitment begins: July 30, 2021.

Approximate date when recruitment will be completed: October 31, 2022.

#### Authors’ contributions {31b}

ARZ is the principal investigator; he conceived the study, sought funding, directed the proposal, and developed the protocol. FAB is the principal investigator; he collaborated in designing the study and developing the intervention protocol. NZA contributed to the design of the study, project and manuscript writing, and upcoming implementation of the protocol. AHR contributed in the study design and contributed drafting and reviewing the manuscript. VCM contributed to the design of the study, project writing, and upcoming implementation of the protocol. All authors read and approved the final manuscript.

## Supplementary Information

**Additional file 1.**

## Abbreviations

PD: Parkinson’s disease
AMPS: Automated mechanical peripheral stimulation
GEX: Exercise group
GAMPS: AMPS group
HRV: Heart rate variability
LF: Low frequencies
HF: High frequencies
SBP: Systolic blood pressure
HR: Heart rate
HO: Orthostatic hypotension
Vo2: Maximum oxygen uptake
VAS: Visual analog scale
BDNF: Brain-derived neurotrophic dactor
PDQ-39: The Parkinson’s Disease Questionnaire
TUG: Timed up and go
CESFAM: Family Health Center
CPET: Cardiopulmonary exercise testing
VCO2: Carbon dioxide output
VE: Ventilation
RER: Respiratory exchange ratio
EGG: Electrocardiogram
